# Development trends of etiological research contents and methods of noncommunicable diseases

**DOI:** 10.1002/hcs2.69

**Published:** 2023-10-09

**Authors:** Dafang Chen, Yujia Ma, Han Xiao, Zeyu Yan

**Affiliations:** ^1^ Department of Epidemiology and Biostatistics, School of Public Health Peking University Beijing China; ^2^ Key Laboratory of Epidemiology of Major Diseases (Peking University), Ministry of Education Peking University Beijing China

**Keywords:** noncommunicable diseases, multimorbidity, etiology, systems perspective, network, life course

**Abbreviation**
NCD: noncommunicable disease

## 1 BACKGROUND

Noncommunicable diseases (NCDs) are a significant public concern, greatly impacting the economic and social development in China. In 2019, NCDs accounted for a staggering 88.5% of total deaths in China, with cardiovascular diseases, cancer, chronic respiratory diseases, and diabetes—the four major chronic diseases—contributing to a premature mortality rate of 16.5% [1]. The complexity of NCDs arises from the involvement of multiple genetic and environmental factors that interact in intricate ways. The complexity is characterized by a multitude of interactions among genes, proteins, and metabolic pathways throughout the various stages of life. Furthermore, these interactions demonstrate time‐dependent specificity during the different phases of the life course. Prior research on the etiology of NCDs tended to focus on “specificity,” which overlooked the concept of “universality.” Studies are often conducted from one risk factor, one disease, or one dimension, leading to an insufficient understanding of NCD etiology and less than satisfactory outcomes in prevention and control efforts. Therefore, the aim of this review is to highlight and propose a new trend in NCD etiology research, considering the research focus and research methodology.

## 2 RESEARCH FOCUS: FROM SINGLE DISEASE TO COMORBIDITY PATTERNS AND DISEASE TRAJECTORIES

The relationships among NCDs are intricate, and patients often show distinct patterns of multiple diseases, reflecting population heterogeneity in comorbidity. The study of comorbidity patterns among populations affected by NCDs can offer valuable insights for developing effective prevention and management strategies. In a retrospective study by Jansana et al. [2] using electronic health records, five multimorbidity clusters were identified among breast cancer survivors in Spain; notably, the “musculoskeletal and cardiovascular disease” pattern showed a significantly higher risk of mortality than other NCDs. Advancements in computational science contribute to the emergence of network analysis based on graph theory as a powerful tool for understanding the complexity of comorbidity from a holistic and systemic perspective. Graph theory in network analysis facilitates the construction of comorbidity networks in which disease status is represented as nodes and risk associations are shown as edges, thereby visualizing the co‐occurrence of diseases in a concise and intuitive manner. Such topological approaches enable the prioritization of disease severity and identification of the core disease within a comorbidity network. Furthermore, network clustering techniques have been applied to identify specific comorbidity patterns in NCDs. However, cautiousness in interpreting the identified patterns is essential because some network topology indexes may lack practical significance. The challenge in interpreting the identified patterns can be addressed by considering association rules. Typically, association rule mining is used to identify comorbidity patterns, and network analysis is used to visualize and determine the core diseases within a comorbidity network. For example, Hernández et al. [3] discovered several comorbidity patterns in Irish adults using association rules and subsequently found that high cholesterol, hypertension, and arthritis had the highest number of associations with other medical conditions by network analysis, designating them as the core diseases in the comorbidity network.

The development of NCDs is a prolonged and gradual process characterized by the accumulation of risks over time. The intricate variations during disease progression mean that patients may have different trajectories leading to the same disease pattern. It is crucial to consider the temporal characteristics of the progression of each disease component, even within a specific comorbidity pattern. Identifying disease trajectories at the population level is vital for preventing comorbidity among specific NCD populations and provides essential epidemiological evidence for understanding the etiology of comorbidity, making comorbidity trajectory research a current research hotspot. Jensen et al. [4] conducted a discovery‐driven analysis of temporal disease progression patterns using data from an electronic health registry that covered the entire population of Denmark. They identified 1171 significant trajectories and grouped them into patterns centered on key diagnoses, such as chronic obstructive pulmonary disease and gout, which was critical to disease progression and early diagnosis to mitigate adverse outcomes. Comorbidity trajectory research in the general population poses challenges in study design, data analysis, and result interpretation, and therefore such research is often carried out in populations with a specific disease, thereby simplifying the design, data analysis, and result interpretation. For example, Jeong et al. [5] investigated type 2 diabetes using population‐wide claim data in a nested case‒control study design. They constructed time‐dependent type 2 diabetes trajectories and formed a comorbidity development network of patients with type 2 diabetes, then calculated the relative risk of progression from type 2 diabetes to other diseases. Several less‐reported comorbidities, such as depression and hearing impairment, as well as time‐critical associations between type 2 diabetes and other diseases, were discovered in the Jeong et al. [5] study. Their findings were beneficial for improving disease management for patients with type 2 diabetes.

Comorbidity of NCDs suggests there may be shared genetic architecture and environmental risk factors among these conditions. At the exposome level, the shared risk factors for comorbidity can be classified as: (1) general external exposure, including climate, social, economic, and psychological factors; (2) specific external exposure, such as occupational factors and individual environmental factors, such as lifestyle behaviors, dietary preferences, and medical interventions; and (3) internal exposure to endogenous substances generated by pleiotropic effectors at the molecular, cellular, tissue, and organ levels [6, 7]. For metabolic disorders, Guo et al. [8] proposed that multiple core pathological processes, including neuroendocrine disruption, insulin resistance, oxidative stress, chronic inflammatory response, and gut microbiome dysbiosis, interconnect and contribute to the onset and progression of metabolic diseases, forming a “multiple hit” scenario. Identifying potential comorbidity mechanisms and shared environmental risk factors and implementing rational preventive measures are effective strategies to reduce the disease burden of NCDs. Pietzner et al. [9] integrated electronic medical records with plasma metabolomics data to construct an “NCDs–clinical risk factors–metabolites” network and identified shared pathways among obesity, smoking, impaired glucose homeostasis, inflammation, lipoprotein metabolism, liver function, and kidney function, showing the potential for “network for comorbidity prevention” approaches. Similarly, Li et al. [10] explored pleiotropic drug targets between heart failure and five prevalent chronic diseases (diabetes, obesity, chronic obstructive pulmonary disease, chronic kidney disease, and obstructive sleep apnea) using public databases. They found that the PI3K/AKT pathway played a crucial role and identified potential drugs, such as sodium‐glucose cotransporter‐2 inhibitors, IL‐1β inhibitors, and metformin, which could be used simultaneously by network analysis. A “disease–genetics–environment” network can help researchers (1) identify shared pathogenic pathways among important comorbidities and provide evidence for investigating underlying mechanisms and potential therapeutic strategies for comorbid conditions and (2) identify modifiable environmental factors associated with comorbidities and offer feasible interventions. These two objectives are key for the development of research on comorbidity patterns in NCDs.

## 3 RESEARCH METHODOLOGY: FROM SINGLE OMICS TO TRANS‐OMICS DYNAMIC NETWORKS, FROM SPECIFIC LIFE STAGES TO THE LIFE COURSE

The “omics” technologies that were inspired by the Human Genome Project have brought about a paradigm shift in research and provided crucial technical support for the concept of holism. This transformative development disrupts the traditional fragmented approach of the conventional “single‐factor versus single‐outcome” paradigm and moves it to the more comprehensive “multi‐factor versus multi‐phenotype” paradigm. This profound shift in perspective expands the scope of research, allowing organisms to be studied holistically and revolutionizing the understanding of life from a simplistic viewpoint to one that acknowledges its inherent complexity [11].

The etiology of NCDs exhibits remarkable adaptability and self‐organization, and has three main attributes. (1) Pleiotropy, where a single molecule can give rise to multiple phenotypes. Hu et al. [12] proposed five models to explain the mapping between genotype and phenotype, which provided a theoretical basis for studying NCD etiology from a pleiotropic perspective. (2) Robustness, where the original function of a molecule is maintained under internal and external perturbations. The interactions are often governed by nonlinear and dynamic control, whereas specific proteins, such as chaperones, act as adaptive mechanisms to buffer the impact of disturbances [13, 14]. (3) Rewiring, the inherent restructuring of interactions between biological units in response to conditional change, where adaptive modifications in intrinsic interactions are involved. These distinct attributes of NCD etiology necessitate a comprehensive analysis from a multilevel and multifactorial perspective. Technological limitations meant that simplistic “one‐versus‐one” approaches were used to study the etiology of NCDs; however, the newer omics technologies have helped to overcome these limitations and allowed trans‐omics network analysis to emerge as a promising approach [15]. In trans‐omics analysis, a group of biological molecules or phenotypes is treated as a single variable that is integrated as an information layer, forming a multilevel structural database. This approach enables an objective and comprehensive reconstruction of the intricate network that connects the human genome, exposome, and phenome within the human body. Given the complexity of the network, biological network models have become the preferred choice [16]. Such models abandon the singular perspective of studying disease etiology from a single molecular or omics level and instead use bioinformatics and computational techniques to discover interactions between molecules, thereby establishing high‐dimensional internal connections between different types of biological data layers. This approach results in the formation of a complex molecular information network and aligns with the principles of systematic biology [17]. Consequently, identifying pathogenic pathways based on trans‐omics data and constructing etiological networks have become indispensable in unraveling the underlying causes of NCDs [16, 18, 19]. Bodein et al. [20] made full use of longitudinal data from transcriptomics, proteomics, and metabolomics to construct single omics networks, then used network propagation via a random walk to establish regulatory networks between multiple omics layers. By identifying inter‐omics interactions that are not captured by single omics analysis, they discovered two core dynamic biological clusters that connected the etiological network of diabetes with renal tubular acidosis and restless leg syndrome. This breakthrough discovery provides new insights into the potential mechanisms and interactions underlying the onset and progression of diabetes.

Network comparison is essential to obtain statistical evidence of pathogenic networks and pathways. There are two typical analysis strategies for network comparison. (1) Hypothesis‐driven strategy, where a comprehensive understanding of the physiological, biochemical, and pathological mechanisms of the disease of interest is required. Based on a priori understanding from previous cellular experiments, animal studies, or omics analyses, a reasonable hypothetical pathogenic network/pathway is outlined in advance. Subsequently, intergroup differences and effects of the network/pathway nodes are examined at the population level to assess the validity and practicality of the initial hypothesis‐based pathogenic network/pathway in the population. (2) Data‐driven strategy, where high‐throughput omics markers are acquired at the population level without any predefined hypothesis. Systematic biology methods are used to construct a network connecting exposure factors, biomarkers, and disease endpoints. Intergroup differences and effects of the network/pathway are evaluated at the population level and used to provide a basis for further experimental validation, drug target identification, and the development of prevention or treatment measures [21]. Ji et al. [22] proposed a powerful score‐based statistical test (NetDifM) to measure group differences in weighted biological networks. They successfully captured differences in gene expression networks between patients with ovarian cancer and healthy controls and identified pathogenic PI3K‐AKT signaling pathways, Notch signaling pathways, and their downstream subnetworks.

In etiological research on NCDs, the dynamic attributes of complex biological systems necessitate temporality and high dimensionality jointly, which indicates the need to investigate the metabolic characteristics of disease occurrence and development across the whole life course. Currently, NCD research has focused mainly on adults; for example, the Framingham study of a population aged 28–74 years [23], the UK Biobank study of a population aged 37–85 years [24], and the China Chronic Disease Prospective study of a population aged 30–79 years [25]. However, the Developmental Origins of Health and Disease theory proposes that maternal nutrition and environmental exposures during pregnancy may affect the risk of the offspring developing NCDs in adulthood [26, 27]. This proposal suggests that etiological research on NCDs should transition from a stage‐specific perspective predominantly focused on adults to a life course approach that encompasses pregnancy, childhood, adolescence, youth, middle age, and old age to identify risk factors associated with the development of NCDs and other health outcomes across the entire lifespan, known as life course epidemiology [28].

Life course epidemiology consists mainly of a risk accumulation model and a critical period model. The risk accumulation model assumes that risk factors, such as environmental exposures, socioeconomic status, and behavioral factors, independently or synergistically have long‐term effects on health. Thus, this model focuses on the accumulation and clustering of exposures because diseases are associated not only with individual exposure but also with household exposure and socioeconomic status [28, 29]. The critical period model emphasizes that biological programming during critical developmental periods may be modified by later physiological or psychological stress [28, 29]. Trajectory analysis is a commonly used longitudinal data processing method in life course epidemiology. Trajectory analysis methods are used to fit growth trajectories to individual exposure data with repeated longitudinal measurements, identify subgroups with potentially different growth trajectories within a population, describe trends in exposure factor growth curves collectively and individually, and explore the cumulative effects and critical/sensitive periods of exposure on disease occurrence and development by analyzing growth curve parameters [30, 31]. Conventional trajectory analysis methods include growth curve fitting based on Z scores, multilevel modeling, group‐based trajectory modeling, and latent class mixed effects models [32, 33]. Zhang et al. [34] performed life course trajectory analysis and mediation analysis to quantify the life course cumulative burden of childhood to adulthood obesity and showed that the adverse effects of obesity on cardiovascular health began in childhood and accumulated over the life course. This study provides new evidence for the early‐life origins of cardiovascular disease and has significant implications for formulating early prevention strategies and measures related to obesity‐associated atherosclerosis.

In summary, previous research on the etiology of NCDs, despite integrating information from multiple omics, has focused mainly on “one‐versus‐one” associations between single molecular biomarkers and the occurrence of a single disease (specificity). Regarding the causal relationships between diseases and health, Professor Chen noted that “Any study on causal relationships is, in fact, extracted from a complex, interdependent network of relationships, and represents a relationship which we conceive may exist” [35, 36]. Therefore, research on NCD etiology should strive to elucidate the complex and interdependent underlying network. Trans‐omics causal network studies integrate omics data into a holistic system to discover multidimensional, multilevel, and multitime point interactions between trans‐omics networks and phenotype networks. Given our understanding of the characteristics of NCD etiology, we believe that the perspective of research on NCD etiology needs to shift from local to systemic and from single biology to systemic biology. Establishing a complete functional atlas between genes and phenotypes throughout the entire life course, known as a genotype–phenotype map, will be essential for a comprehensive understanding of the etiology of NCDs.

## AUTHOR CONTRIBUTIONS

**Dafang Chen**: Conceptualization (lead); funding acquisition (lead); project administration (lead); supervision (lead). **Yujia Ma**: Investigation (lead); methodology (lead); writing—original draft (lead); writing—review & editing (lead). **Han Xiao**: Investigation (equal). **Zeyu Yan**: Investigation (equal).

## CONFLICT OF INTEREST

The authors declare no conflict of interest.

## ETHICS STATEMENT

Not applicable.

## INFORMED CONSENT

Not applicable.

## Data Availability

Data sharing is not applicable to this article as no datasets were generated or analyzed during the current study. Not applicable because no datasets were generated or analyzed during this study.
